# Shake and Fake: the Role of Interview Anxiety in Deceptive Impression Management

**DOI:** 10.1007/s10869-020-09708-1

**Published:** 2020-08-07

**Authors:** Deborah M. Powell, Joshua S. Bourdage, Silvia Bonaccio

**Affiliations:** 1 Department of Psychology, University of Guelph, Guelph, ON N1G 2W1 Canada; 2 Department of Psychology, University of Calgary, Calgary, Canada; 3 Telfer School of Management, University of Ottawa, Ottawa, Canada

**Keywords:** Employment interview, Interview anxiety, Impression management, Self-presentation, Extraversion, Honesty-humility

## Abstract

We investigated whether anxiety about self-presentation concerns during interviews (i.e., interview anxiety) is associated with applicants’ use of deceptive impression management (IM) tactics. We examined the relationship between interview anxiety and deceptive IM, and we examined whether the personality traits of honesty-humility and extraversion would be indirectly related to deceptive IM through interview anxiety. Participants (*N* = 202) were recruited after an interview for a research assistant position. Interview anxiety scores were positively related to deceptive IM. Furthermore, there was evidence of a negative indirect effect of honesty-humility on deceptive IM, via overall interview anxiety. Also, extraversion was indirectly associated with deceptive IM through interview anxiety. Results suggest that deceptive IM can be used as a protective mechanism to maintain self-esteem or to avoid the loss of rewards. This paper is the first to examine the role of interview anxiety in interview faking.

**Electronic supplementary material:**

The online version of this article (10.1007/s10869-020-09708-1) contains supplementary material, which is available to authorized users.

Employment interviews are among the most popular selection tools used in organizations (McCarthy & Cheng, 2014; Pulakos, 2005). Because the interview plays such an important role in securing employment, interviewees strive to make a positive impression in front of the interviewer. For example, interviewees may attractively describe past accomplishments, compliment the interviewer, or defend negative aspects of their record; in some cases, they may even stretch the truth or fabricate their past experiences or fit with the organization (Levashina & Campion, 2007). These latter interview behaviors, such as exaggerating or fabricating experiences or fit, have been labeled interview faking, or deceptive impression management (IM). Past research has investigated which interviewees are most likely to engage in deceptive IM and under which circumstances they are likely to do so. This literature has looked at a number predictors of interview faking, including the personality traits of extraversion (e.g., Bourdage, Roulin, & Tarraf, 2018; Weiss & Feldman, 2006) and honesty-humility (e.g., Buehl & Melchers, 2017; Roulin & Bourdage, 2017), and situational factors, such as the competitiveness of the hiring situation (e.g., Ho, Powell, Barclay & Gill, 2019).

Despite this increased research interest in deceptive IM, our understanding of the more proximal factors impacting deceptive IM, and the root of such behavior, remains unclear. However, one important theoretical proposition that could inform our understanding of deceptive IM is that IM may be a self-protective mechanism (Schlenker & Leary, 1982). In accordance with this idea, it may be that concerns about being able to create the desired impression, or about being perceived negatively, are sources of anxiety for some interviewees. For example, interviewees may worry that the image they project is not the “correct” one, or that they will not do a good job of portraying a particular image due to a lack of interpersonal skill or experience. In this paper, we review Schlenker and Leary’s (1982) conceptualization of self-presentation concerns as an underlying cause of social anxieties. We then examine employment interview anxiety, a specific type of social anxiety, and its relationship with deceptive IM use.

We strive to make two novel contributions to the employment interview literature. Guided by the theoretical proposition that IM may be a self-protective mechanism (Schlenker & Leary, 1982), we investigate the role of interview anxiety in deceptive IM. First, we provide a preliminary understanding of how interview anxiety is associated with deceptive IM use, and the idea that anxiety about self-presentation concerns in the interview is associated with applicant use of IM tactics. Second, we investigate interview anxiety as a more proximal mechanism that may help explain the relationship between personality and deceptive IM.

By investigating the link between interview anxiety and deceptive IM, we contribute to a more nuanced understanding of applicants’ motivation to engage in interview faking. This is an important endeavor: while interview anxiety has been a critical construct in the interview literature at large, aside from some very limited mention (Levashina & Campion, 2006), it has not been theoretically or empirically integrated into our understanding of interview faking. Practically, understanding the proximal mechanisms associated with interview faking also provides potential insight into how to reduce faking. As such, in pursuing this investigation, we connect two important research traditions in employment interview: IM (Levashina & Campion, 2007) and interview anxiety (McCarthy & Goffin, 2004). We begin with a discussion of how and why interview anxiety and IM may relate to one another.

## Self-Presentation and Social Anxiety

Whether in the employment interview or any other social situation, self-presentation is the “attempt to control images that are projected in real or imagined social interactions” (Schlenker, 1980, p. 6). The goal of self-presentation is to generate particular images of the self, and thus influence how one is perceived and evaluated by an audience. Different job candidates may have different self-presentation goals during an interview. For example, applicants might try to present themselves in ways that will achieve self-verification (i.e., being seen accurately by the interviewer; Moore, Lee, Kim, & Cable, 2017), or that will result in being liked or respected by the interviewer (e.g., Kristof-Brown, 2000). The type of image that candidates try to create depends on what goals they are trying to achieve. Regardless of a candidate’s specific goals, theory and empirical findings indicate that it is particularly important to be viewed as likeable/warm and competent (e.g., Amaral, Powell, & Ho, 2019; Ferris & Judge, 1991; Leary & Kowalski, 1990) and honest (Jansen et al., 2012; Jones & Pittman, 1982). Therefore, during the interview, self-presentation is often aimed at fostering these images.

Although individuals may want to project a particular image, they may (a) be uncertain about how to go about doing so, or (b) think they will not be able to project the types of images that will produce preferred reactions from others (e.g., they may want to be seen as competent but doubt they will be). That is, despite their desire to project a particular image, some people may believe they will not achieve the preferred image-relevant reaction from others. These two conditions are likely to generate social anxiety. Indeed, Schlenker and Leary (1982) argued that there is a common denominator to all social anxieties—concerns over self-presentation. In line with this argument, Leary and Kowalski (1990) note that two of the primary self-presentational motives that drive individuals to try to portray particular images are to maintain their own self-esteem, and when valued social and material rewards are on the line. The interview is one such situation where these components are strongly present, but some individuals may lack (or feel that they lack) the means to be viewed positively by others, or feel that their current self is discrepant from what an evaluator may value.

## Interview Anxiety as a Specific Type of Social Anxiety

Broadly speaking, social anxieties result from “the prospect or presence of personal evaluation in real or imaged social situations” (Schlenker & Leary, 1982, p. 642). It is the potential for interpersonal evaluation that distinguishes social anxiety from other types of anxiety, such as anxieties about physical danger. People’s perceived inability to deal successfully with the evaluative nature inherent in social interactions is what triggers social anxiety. Applied to the interview, this implies that interview anxiety may be, in part, a reaction to negative self-presentation concerns in a highly evaluative context.

As a selection tool, interviews require a sustained interaction between job applicants and employer representatives. During this interaction, job applicants must convey competence, interest in, and fit with, the organization, and ensure that the interviewer judges them accordingly (Huffcutt, Conway, Roth, & Stone, 2001). In other words, employment interviews are evaluative, high-stakes situations of a highly social nature (McCarthy & Goffin, 2004). As a result, many job applicants experience anxiety about the interview process (Heimberg, Keller, & Peca-Baker, 1986; Powell, Stanley, & Brown, 2018).

McCarthy and Goffin (2004) define interview anxiety as “feelings of nervousness or apprehension that are relatively stable within job applicants across employment interview situations” (p. 616). Interview anxiety fits under a broader category of social anxieties; that is, anxieties that are activated in the presence of other people (Schlenker & Leary, 1982). Other social anxieties include anxiety about giving speeches or being in a room full of strangers.

McCarthy and Goffin (2004) outlined five dimensions of interview anxiety; each dimension focuses on feelings of nervousness or apprehension around a specific aspect of the interview. Communication anxiety focuses on feelings of nervousness surrounding verbal communication, nonverbal communication, and listening skills. Appearance anxiety centers on physical appearance (e.g., concerns about body image, or dress style). Social anxiety focuses on being able to effectively use appropriate social behaviors (e.g., using a correct handshake, being able to build rapport).^1^ Performance anxiety is a fear of failure, or a concern over the outcome of the interview. Finally, behavioral anxiety involves the autonomic reactions one’s body has to the interview situation (e.g., fast heartbeat, sweaty palms). Although the five interview anxiety dimensions are conceptually distinct, most research has focused on overall interview anxiety (e.g., Feiler & Powell, 2013; Gong, Li, Zhang, & Rost, 2016). These dimensions of interview anxiety, as conceptualized by McCarthy and Goffin (2004), underscore the interpersonal, social, and evaluative nature that is characteristic of interviews.

## IM Tactics and Interview Anxiety

For many people, experiencing self-presentation concerns in an interview can be a source of social anxiety. In the present study, we propose that one response to such social anxiety is for applicants to engage in impression management behaviors (IM). IM has been defined as “job candidates’ attempts to control and determine the image interviewers’ form of them regarding their behaviors, motivation, and other attributes” (Levashina & Campion, 2006, p. 299). IM tactics are commonly categorized as being self-focused (e.g., promoting one’s skills and abilities in an effort to appear competent) or other-focused (e.g., demonstrating values one has in common with another person to appear likable). In addition, IM tactics can be considered honest (being sure to promote skills one is very good at) or deceptive (exaggerating the actual level of one’s skills). In the present study, we focus on deceptive IM tactics, as these may be particularly salient for managing one’s self-presentational concerns in the interview when applicants perceive a discrepancy between their current and desired self-image, as discussed below.^2^

There are a number of theoretical reasons why deceptive IM would be a response to experiencing interview anxiety. Specifically, when individuals experience self-presentation concerns, and believe that presenting their true selves in an interview would not be in their best interest, they might be tempted to engage in such IM behaviors. Applicants may have to choose between multiple images of themselves, or perhaps even a slightly exaggerated or false image of themselves. Indeed, Leary and Kowalski (1990) argued that the impression motivation process is affected by the discrepancy between the individual’s current image and the image he or she desires to convey. Similarly, these authors note that individuals are motivated to convey a desired image that the target values—in the case of an interview, competent, likeable/warm, and honest—and avoid undesired images. Finally, these authors note that when individuals are particularly concerned about social and material rewards, which are both at stake in the job interview, they may be more motivated to impression manage. In short, several of the same processes that are thought to drive anxiety—evaluative contexts, worries about being perceived as inadequate—are also central to our understanding of IM. As such, it may be that self-presentation concerns during the interview (i.e., interview anxiety) could actually be an underlying mechanism associated with people’s choice to engage in IM tactics during the interview.

There has been little research that has directly set out to investigate the relation between interview anxiety and IM in interviews; however, there have been at least two studies that have measured and reported results for both interview anxiety and deceptive IM. In a sample of students completing mock interviews under a variety of different conditions, Law, Bourdage, and O’Neill (2016) looked at the performance dimension of interview anxiety and reported that “it appears that those who used deceptive IM reported significantly higher performance anxiety, with an average correlation of 0.35” (p. 7). Similarly, Charbonneau and Powell (2018) reported a correlation of 0.33 between interview anxiety and an overall measure of deceptive IM. Although the correlations were reported in both studies, this relation was not the research question of interest of either study. As such, the authors did not speculate on why interview anxiety and deceptive IM might be related. In the present study, based on the theoretical link between social anxiety and IM as a means to cope with self-presentation concerns, we seek to replicate the finding of a positive relation between interview anxiety and deceptive IM. As such, we propose the following hypothesis.

H1: There will be a positive relation between interview anxiety and deceptive IM.

## Personality and Self-Presentation

The expected positive relation between interview anxiety and IM might help elucidate the relation between personality traits associated with self-presentation concerns and the use of deceptive IM. Indeed, while there have been a number of studies on the topic of personality predictors of deceptive IM in recent years (e.g., Buehl & Melchers, 2017; Levashina & Campion, 2007; Roulin & Bourdage, 2017), works investigating the mechanisms involved in this association have been much sparser. In terms of understanding what personality variables are most likely to play a role in this process, we turn to Schlenker and Leary (1982). According to these researchers, people will be interpersonally secure in social settings when (a) they do not have the goal of creating a particular impression on others and hence are not immediately concerned about others’ evaluative reactions, or (b) they are attempting to create a particular impression and believe they will be successful in doing so. These two mindsets may be captured by stable personality traits, in particular honesty-humility and extraversion.

### Honesty-Humility

People higher in honesty-humility are honest, sincere, and straightforward, and do not try to take advantage of others for their own gain (Lee & Ashton, 2004). Within the interview context, honesty-humility has been found to be the personality variable most robustly related to IM, as a negative predictor of deceptive IM. In fact, many recent studies report correlations in the − 0.20s to − 0.40s (for a review, see Melchers, Roulin, & Buehl, 2020). This consistent negative relation is likely reflective of the tendency of individuals low in honesty-humility to adopt an exploitative or manipulative approach to others, whereas those high in honesty-humility emphasize fairness, cooperation, and transparency (Ashton & Lee, 2007). Consistent with this argument, research indicates that individuals low in honesty-humility are more likely to view faking as wise and useful (Bourdage, Schmidt, Wiltshire, Nguyen, & Lee, 2019; Buehl & Melchers, 2017).

Individuals low in honesty-humility may be particularly likely to have self-presentation concerns. For instance, past research has suggested that individuals will want to portray themselves as honest in an interview context, which will be difficult for those low in honesty-humility. Indeed, Jones and Pittman (1982) note that “aside from wanting others to think of us as competent and likable, we usually want them to think of us as morally worthy: honest, generous, self-sacrificing” (p. 247). Similarly, studies of recruiters indicate that they consider dishonest behavior as very inappropriate (Jansen et al., 2012), and perceptions of dishonesty negatively impact interview performance ratings (Roulin et al., 2015). Given all of this, individuals who are high in honesty-humility will experience less discrepancy between their actual and desired image. On the other hand, those who are low in honesty-humility will likely view themselves as being more discrepant from the ideal image, and therefore may experience more concerns about their current image. These self-presentation concerns might give rise to interview anxiety.

In addition, an individual’s honesty-humility should impact the extent to which they are worried about doing well in the interview. A primary driver of impression motivation is the desire to obtain social and material rewards (Leary & Kowalski, 1990). Such a concern would be tied to different components of anxiety, such as performance anxiety—a concern about doing poorly. Interestingly, individuals high in honesty-humility tend to be less concerned with gaining things of instrumental value, whereas those low in honesty-humility tend to be very motivated by status, power, and material rewards (Lee et al., 2013). Moreover, given the association between low honesty-humility and narcissism (Lee et al., 2013), individuals low in honesty-humility may see a negative evaluation as an ego threat. Conversely, we predict people high in honesty-humility are likely to experience less self-presentation concerns, and thus less interview anxiety, because they are less worried about the potential material rewards.

Consistent with this rationale, Law et al. (2016) reported a correlation of − 0.15 between honesty-humility and interview performance anxiety. Because people high in honesty-humility are straightforward in social interactions, and tend to act consistently across situations (e.g., Hilbig & Zettler, 2009), there is less need to decide between multiple versions of oneself during self-presentation; high honesty-humility individuals will simply present themselves as they are—not as they guess another person wants them to appear. Therefore, interview anxiety could be an underlying mechanism to help explain the relation between honesty-humility and deceptive IM. If people higher in honesty-humility experience less discrepancy between their current image of themselves and their desired image during an interview, and are less worried about being evaluated negatively, they consequently may experience less anxiety, and feel less need to engage in techniques such as image protection and image creation as methods to control the image they put forth in an interview. Conversely, people low in honesty-humility tend to be very status-oriented. As such, these individuals may be particularly concerned about the ability to create a positive impression and accrue the rewards associated with successful IM. This explanation is in line with Leary and Kowalski’s (1990) argument that two primary self-presentational motives that drive individuals to engage in IM are maintenance of self-esteem and having valued social and material rewards on the line.

H2: Honesty-humility will be indirectly related to deceptive IM through interview anxiety such that honesty-humility will be negatively related to interview anxiety, which in turn will be positively related to deceptive IM.

### Extraversion

Interviews are an “interactional process that involve a social exchange” (McCarthy & Goffin, 2004, p. 611). As such, a second personality trait that may be relevant to interview anxiety is extraversion. People higher in extraversion are more assertive, confident, and comfortable in social situations (Lee & Ashton, 2004; Wilt & Revelle, 2017). In addition, individuals high in extraversion value social-oriented status (Ashton & Lee, 2007). As such, while they are likely motivated to create a positive impression, they may be less worried about their ability to portray their desired image. These characteristics of extraversion are consistent with Schlenker and Leary’s (1982) explanations of when people are likely to feel interpersonally secure in social settings—when they believe they will be successful. Following this logic, extraverts would likely believe they would be more successful in an interview context. Indeed, extraverts largely seem to be at an advantage in the interview, with meta-analytic results indicating that interviews are in large part measuring extraversion (Salgado & Moscoso, 2002).

There is some support for a negative relation between extraversion and interview anxiety, although the research is limited. For example, McCarthy and Goffin (2004) reported positive correlations between public self-consciousness and interview anxiety as well as low self-confidence and interview anxiety—traits that are similar to the low end of extraversion. Similarly, Cook, Vance, and Spector (2000) found a negative relation between extraversion and trait anxiety in the context of job interviews. There is also some unpublished work that has found negative relations between extraversion and interview anxiety (*r* = − 0.33 to − 0.24; Schneider, 2015). Although the research is limited, extraversion—a trait that describes confidence in social situations—does appear to be negatively related to interview anxiety.

Research also indicates that extraversion tends to positively relate to more honest attempts at IM (e.g., Bourdage et al., 2018; Kristof-Brown et al., 2002.) In contrast, the association between extraversion and deceptive IM is less robust, being either positive (Weiss & Feldman, 2006; Roulin & Bourdage, 2017), negative (Bourdage et al., 2018), or null for some dimensions (Roulin & Bourdage, 2017). The fact that extraversion tends to be associated with more honest IM and is often unrelated or negatively related to deceptive IM led Bourdage et al. to note that extraverts in general may be more comfortable with their abilities to portray their desired image, whereas more introverted individuals may struggle to create a good impression through legitimate means, leading them to turn to deceptive means in some cases.

Whereas the direct association between extraversion and deceptive IM may on the whole be less robust, it is possible that there is an indirect relationship between these two variables. Specifically, when thinking about anxiety, it is possible that there is a negative indirect relationship between extraversion and deceptive IM through interview anxiety, such that individuals high in extraversion are less likely to engage in deceptive IM because they are less anxious about being able to create their desired impression. Conversely, individuals who are more introverted, in a setting that rewards extraversion, is fundamentally social, and values coming across as warm and likable, may be particularly anxious, and attempt to make up for this discrepancy through the use of deceptive IM.

H3: Extraversion will be indirectly related to deceptive IM through interview anxiety such that extraversion will be negatively related to interview anxiety, which in turn will be positively related to deceptive IM.

## Methods

### Participants and Procedures

Students who applied for a real research assistant position in the winter of 2017^3^ and 2018 were interviewed and invited to participate in this study. Advertisements were posted widely throughout campus to inform students of a research assistant position. Every student who applied to the job received a scheduled interview time and was invited to take part in a study following the interview. The interview was conducted by one of four trained research assistants and consisted of four behavior descriptive interview questions. The interview ratings were used as part of the eventual hiring decision.

After completing the interview, the interviewees were brought to a different room, and they were asked whether they would like to participate in the research study. If they agreed, a researcher went through the consent process with them and set them up on the computer to complete a Qualtrics survey. The survey contained the measures described below and asked the participants to reflect on the interview they had just completed. Of the 237 applicants who interviewed, 202 applicants agreed to be in the study. The mean age was 21.12 (SD = 4.06) and 82.7% were female.

We took several steps to mitigate the risk that participants might feel coerced into participation. First, the professor who ultimately made the hiring decision for the research assistant was never told who did or did not participate in the study, and interviewees were told this. Second, the interviewer never knew who did or did not participate in the study, so it could not have affected their rating, and interviewees were told this. Third, the participants were walked to a different room from where the interview took place, and then, the interviewer left this new room. Once in the new room, interviewees were invited to be in the study by a research assistant. Finally, interviewees were sent the letter of information (i.e., the consent form) about the study by email well in advance so they had time to reflect on the study invitation. In this letter, applicants were told that they would be invited to participate in a study after the interview, information about the nature of the study (e.g., how much time would be required) that participation was voluntary, and participation (or not) would not affect their chances of being hired. This was done to ensure participants did not feel surprised by the invitation, and therefore would not feel compelled to agree to participation. As well, we only recorded a participant number on the survey, and we assured participants that their responses would in no way be connected to their chance of being hired for the position. As a way to try to confirm that these steps had the intended effect (that is, did participants believe us that their participation or not would not affect their chances of getting the job), we asked them “How confident are you that your responses from this questionnaire will be kept confidential?” rated on a 5-point scale ranging from 1 (not at all confident) to 5 (completely confident). The mean was 4.57 (SD = 0.73).

### Measures

#### Deceptive IM

The Interview Faking Behavior-Short scale contains items derived from Levashina and Campion’s (2007) interview faking behavior scale. It contains 16 items that measure image protection, deceptive ingratiation, slight image creation, and extensive image creation (Bourdage et al., 2018). The following sample item for extensive image creation demonstrates the possible extreme nature of interview faking: “I told fictional stories prepared in advance of the interview to best present my credentials” (Bourdage et al., 2018). The four subscales were averaged to create an overall measure of deceptive IM. This measure, and all other measures described below, employed a 5-point scale ranging from 1 (strongly disagree) to 5 (strongly agree).

#### Honest IM

Although we did not have specific hypotheses about honest IM in this study, we included these items to take an exploratory look at the relation between anxiety and these important IM behaviors. The honest interview IM-short scale (Bourdage et al., 2018) was used to measure honest IM. It contains 12 items to measure self-promotion, honest ingratiation, and defensive honest IM (e.g.,: “I made sure to let the interviewer know about my job credentials”).

#### Interview Anxiety

We used McCarthy and Goffin’s (2004) 30-item self-report Measure of Anxiety in Selection Interviews (MASI) to measure interview anxiety*.* We adapted the wording slightly to reflect anxiety about the specific interview participants had just completed (rather than interviews in general). A sample item from this scale is: “I got so anxious while taking the interview that I had trouble answering questions that I know.”

#### Honesty-Humility

We measured honesty-humility using the 10-item honesty-humility scale from the HEXACO personality inventory (Lee & Ashton, 2004, 2006). A sample item is: “I am an ordinary person who is no better than others.”

#### Extraversion

We measured extraversion using the 10-item extraversion scale from the HEXACO personality inventory (Lee & Ashton, 2004, 2006). A sample item is: “In social situations, I’m usually the one who makes the first move.”

## Results

Means, standard deviations, and intercorrelations among variables are presented in Table 1. Consistent with past research, overall interview anxiety was positively correlated with deceptive IM, ranging from *r* = 0.13, *p* = .08 (extensive image creation) to *r* = 0.26, *p* < .001 (image protection). Thus, hypothesis 1 was supported. In addition, we examined the correlations of deceptive IM with different dimensions of interview anxiety. The strongest relations with overall deceptive IM were with social (*r* = 0.30, *p* < .001) and communication anxiety (*r* = 0.26, *p* < .001), although all forms of interview anxiety significantly correlated with overall deceptive IM.

**Table 1:** Means, Standard Deviations and Correlations Between Variables

Variable	*M*	*SD*	1	2	3	4	5	6	7	8	9	10	11	12	13	14	15	16	17	18
1 Gender	--	--	--																	
2. Age	21.12	4.06	.02	--																
3. DIM Slight Image Creation	1.71	.66	-.06	-.11	*(.66)*															
4. DIM Extensive Image Creation	1.24	.44	.01	-.03	.43**	*(.65)*														
5. DIM Deceptive Ingratiation	1.71	.76	.08	-.01	.45**	.29**	*(.78)*													
6. DIM Image Protection	1.45	.51	.05	-.04	.34**	.26**	.41**	*(.42)*												
7. DIM Overall	1.52	.43	.03	-.08	.79**	.62**	.80**	.67**	*(.81)*											
8. HIM Self-Promotion	3.40	.72	.06	.02	.14*	.04	.22**	.10	.19**	*(.77)*										
9. HIM Ingratiation	1.98	.78	.01	.03	.42**	.17*	.76**	.35**	.64**	.42**	*(.73)*									
10. HIM Defensive	2.44	.83	-.14	.04	.24*	.18	.35**	.28*	.37**	.24*	.38**	*(.59)*								
11. IA Communication	2.67	.75	-.04	-.16*	.23**	.17*	.18*	.19**	.26**	-.42**	-.05	-.02	*(.80)*							
12. IA Appearance	2.36	.85	.13	-.13	.12	.08	.14*	.21**	.19**	.03	.03	.08	.23**	*(.82)*						
13. IA Social	2.60	.88	.14*	-.15*	.23**	.13	.25**	.24**	.30**	-.06	.12	.02	.51**	.60**	*(.83)*					
14. IA Performance	3.47	.85	.13	-.22**	.15*	.04	.21**	.18**	.21**	-.15*	.08	-.04	.57**	.48**	.65**	*(.85)*				
15. IA Behavioral	2.61	.79	.11	-.20**	.16*	.07	.20**	.18*	.22**	-.16*	.10	-.16*	.47**	.40**	.55**	.67**	*(.76)*			
16. IA Overall	2.74	.64	.13	-.22**	.23**	.13	.25**	.26**	.30**	-.18**	.08	-.03	.70**	.71**	.85**	.86**	.78**	*(.93)*		
17. Honesty-Humility	3.52	.58	.07	.09	-.25**	-.16*	-.16*	-.26**	-.29**	-.05	-.10	.09	-.17*	-.11	-.10	-.18**	-.05	-.15*	*(.70)*	
18. Extraversion	3.43	.59	-.08	.11	.08	-.01	-.03	-.07	-.01	.12	.12	.05	-.24**	-.23**	-.39**	-.30**	-.30**	-.38**	.06	*(.78)*

*Note.* N = 202 for focal variables. Exceptions are for correlations with age (N = 200), gender (N = 201), and honest defensive Impression Management (N = 78). Reliabilities (alpha) are on the diagonal. For gender, male = 1 and female = 2. DIM = Deceptive Impression Management. HIM = Honest Impression Management. IA = Interview Anxiety.

* indicates *p* < .05. ** indicates *p* < .001.

Regarding the correlations between personality and interview anxiety, honesty-humility was negatively related to overall interview anxiety (*r* = − 0.15, *p* = .03), as was extraversion (*r* = − 0.38, *p* < .001), suggesting that applicants higher in these personality traits report experiencing less interview anxiety. In terms of correlations with specific facets of anxiety, honesty-humility significantly and negatively correlated only with performance anxiety (*r* = − 0.18, *p* = .01) and communication anxiety (*r* = − 0.17, *p* = .02), whereas extraversion negatively correlated with all forms of interview anxiety, with particularly strong relations with social anxiety (*r* = − 0.39, *p* < .001).

**Fig. 1:**
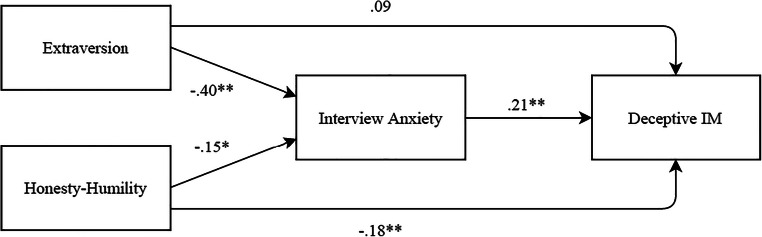
Indirect effect of honesty-humility and extraversion on deceptive impression management through interview anxiety. Values are unstandardized coefficients. Significance tests are based on bootstrap methodology, 5000 bootstrapped samples, 95% confidence intervals. *p < .05, **p < .01. *N* = 202. Unstandardized indirect effect of honesty-humility on deceptive IM is − 0.031 (95% CI [− 0.066, − 0.002]). Unstandardized indirect effect of extraversion on deceptive IM is − 0.083 (95% CI [− 0.142, − 0.039])

Hypotheses 2 and 3 posited that honesty-humility and extraversion would be indirectly related to deceptive IM through interview anxiety. We tested this using the PROCESS macro developed by Hayes (2017). Honesty-humility and extraversion were entered into a single mediation model using model 4. To do so, we followed the recommendations by Hayes (2018, pp. 141–145) to execute PROCESS twice, once by considering honesty-humility as the substantive predictor variable of interest, and including extraversion as a covariate, and once by considering extraversion as the substantive predictor variable, and including honesty-humility as a covariate. Path coefficients for the full model are presented in Fig. 1. First, regarding honesty-humility, the unstandardized indirect path (*b* = − 0.031, 95% CI [− 0.066, − 0.002]) was statistically significant, indicating a negative indirect effect of honesty-humility on deceptive IM, via overall interview anxiety. Thus, hypothesis 2 was supported. However, the direct effect (*b* = − 0.183, 95% CI [− 0.280, − 0.086]) found from honesty-humility to deceptive IM once interview anxiety was included in the model suggests interview anxiety does not fully account for this relationship.

Hypothesis 3 stated that the extraversion would be indirectly related to deceptive IM through interview anxiety. The direct effect (*b* = 0.089, 95% CI [− 0.012, 0.191]) between extraversion and deceptive IM was not significant, either with or without interview anxiety in the model. However, consistent with hypothesis 3, extraversion was indirectly associated with deceptive IM through interview anxiety, with an unstandardized indirect effect of *b* = − 0.083, 95% CI [− 0.142, − 0.039]). Path coefficients for the full model are presented in Fig. 1.

### Exploratory results

Although we did not have any specific hypotheses about the relation between honest IM and interview anxiety, we did find it interesting that overall interview anxiety had a negative relation (*r* = − 0.18, *p* = .009) with honest self-promotion, and near-zero relations with the honest ingratiation (*r* = 0.08, *p* = .28) and honest defensive (*r* = − 0.03, *p* = .81) dimensions of honest IM.

## Discussion

In this study, we found a moderate, positive correlation between interview anxiety and self-reported use of deceptive IM. This finding supported our hypothesis and replicated two past studies that have reported, but had not hypothesized or discussed, the correlations between these two variables (Charbonneau & Powell, 2018; Law et al., 2016). This finding provides important support for an additional mechanism for understanding deceptive IM and suggests that interview anxiety should be integrated into models of deceptive IM. While existing models of deceptive IM in interviews (e.g., Levashina & Campion, 2006; Roulin, Krings, & Bingeli, 2016) and faking on personnel selection tools more broadly (e.g., Goffin & Boyd, 2009; Marcus, 2009; Mueller-Hanson, Heggestad, & Thornton, 2006) have recognized the potential importance of individual difference factors such as self-esteem, public self-consciousness, need for approval, and emotional stability, which may be related to interview anxiety, none of these models specifically addresses the role that interview anxiety may play in our understanding of interview faking. For instance, anxiety may be one factor that positively impacts an applicant’s willingness to use IM (Levashina & Campion, 2006). This is consistent with the idea that deceptive IM may not be something applicants do comfortably; rather, deceptive IM may be more of a self-protective mechanism (Schlenker & Leary, 1982), potentially used to make up for some discrepancy between applicants’ current perceived skills or fit and the desired impression they want to convey (Leary & Kowalski, 1990).

In line with this reasoning, we also found that interview anxiety can help explain the relationship between low honesty-humility and deceptive IM and low extraversion and deceptive IM—two traits that are likely associated with self-presentation concerns. These findings help elucidate the questions of who engages in IM and why they do so. Deceptive IM may be motivated by a protective mechanism to maintain self-esteem when people have self-presentation concerns (Leary & Kowalski, 1990). We found support for the idea that interviewees characterized by high honesty-humility may be less anxious about the interview (perhaps because there simply is less discrepancy between who they are and the desired image they want to present), which may result in less use of deceptive IM. Conversely, individuals who are low in honesty-humility may have more discrepancy between current and desired image, and be more instrumentally motivated to do well in the interview, driving some anxiety. Although this relationship is smaller than that observed with extraversion (discussed below), it is consistent with previous research in an experimental setting (Law et al., 2016). We also found support for the idea that those who are more likely to be confident in their ability to present their desired image (high extraversion) experience less interview anxiety and are subsequently less likely to use deceptive IM tactics. It may be that interviewees high in extraversion believe they have the required skills to present their desired images in interview settings, where extraversion and sociability are valued traits (Huffcutt et al., 2001; Salgado & Moscoso, 2002).

In addition to looking at relations between personality traits and overall interview anxiety, we also looked at relations with the facets of interview anxiety scale, to provide further insight into how personality may relate to interview anxiety. Honesty-humility was most significantly negatively correlated with performance anxiety. This finding is consistent with the idea that individuals low on honesty-humility tend to be more status and success-oriented, and may be particularly anxious about doing better than other people and securing desired outcomes. On the other hand, those low on extraversion seem to experience interview anxiety in a number of diverse dimensions, although the particularly strong correlation with social anxiety indicates that these individuals are most worried they will be perceived as socially awkward or unlikable. Overall, this study contributes to the limited knowledge of personality correlates of interview anxiety, and indicates that individuals who differ on the personality traits of honesty-humility and extraversion may have unique anxieties, and that these anxieties are associated with faking.

### Strengths and Limitations

The strength of the current study is that we assessed job applicants interviewing for a real job, rather than participants engaged in mock interviews. This is a notable advantage, especially in research focused on interview anxiety. While anxiety can also be felt in mock interviews (e.g., Feiler & Powell, 2016), a field setting allows for greater external validity. On the other hand, because the applicants were undergoing real interviews, they may have been hesitant to self-report their use of deceptive IM. To mitigate this risk, we implemented several safeguards as previously explained in the “Methods” section. Other field investigations of deceptive IM have used similar safeguards (e.g., Roulin & Bourdage, 2017).

While the strength of the study was assessing job applicants, we should acknowledge the context of our study. The interviewees were competing for a research assistant position, and a majority of the interviewees were young female university students. Future research should seek to extend our findings by focusing on a wider range of jobs and industries and more diverse kinds of job seekers. Indeed, studies including more experienced job seekers report lower levels of deceptive IM (Roulin, Bangerter, & Levashina, 2014). As Melchers et al. (2020) suggest, more experienced applicants may have less need to engage in these behaviors. In terms of our theoretical framework, this might mean that more experienced applicants have a narrower discrepancy between their current and the desired image they wish to project. Future research could investigate this possibility.

A second limitation of our design is the reliance on self-report measures. Indeed, IM in interviews is a line of inquiry that has relied on self-report almost by necessity—observers are not accurate judges of interviewees’ reliance on IM—and there are no measures other than self-report to assess the dimensions of honest and deceptive IM (Melchers et al., in press; Roulin et al., 2015). Similarly, McCroskey (1984) has argued that self-report measures are the most appropriate measure when they are related to affect or perception (as is the case with interview anxiety), and when the respondent has no reason to expect negative consequences from their responses—conditions which we tried to set up in this study.

It is also worth noting that all study measures were assessed at the same time point, so causal direction of the relations could not be tested. It could be the case, for example, that choosing to engage in deceptive IM in the interview then leads to the experience of anxiety during the interview. However, if it were the case that deceptive IM leads to interview anxiety, we might expect that the most extreme form of deceptive IM (extensive image creation) would correlate most strongly with interview anxiety. Instead, the weakest correlations with interview anxiety were found with extensive image creation. This pattern of correlations, where interview anxiety is most strongly related to slight image creation, image protection, and deceptive ingratiation, which are more “mild” and spontaneous forms of faking (in contrast to more “severe” and planned faking; Fell, König, & Kammerhoff, 2016), is consistent with Schlenker and Leary’s (1982) argument that concern over self-presentation (i.e., when trying to create a particular impression) is the underlying cause of social anxieties. However, with the current research design, we cannot determine whether or not anxiety is an “underlying cause” of deceptive IM. Interviewees may be deceptive because they are anxious, or anxious because they were deceptive and are concerned about the consequences. Measuring interview anxiety both before and after the interview would be helpful in an attempt to better understand cause and effect.

### Future Research Directions

We found it interesting in this study that interview anxiety was positively correlated with deceptive IM and negatively correlated with honest self-promotion. This could be because individuals who engage in honest self-promotion tend to be more extraverted and more experienced (Bourdage et al., 2018) and hence may have a lower discrepancy between their true and desired identities. As such, individuals who are going into the interview feeling that they have the capacity to create an authentic positive impression are less likely to feel anxious, and subsequently more willing and able to engage in honest self-promotion. In the current study, the correlation between honest self-promotion and extraversion was positive (*r* = 0.12, *p* = .09), but non-significant, which differs slightly from the findings of Bourdage et al. (2018), who found a correlation of *r* = 0.17, *p* < .05. Interestingly, interview anxiety has been found, in past research, to be negatively associated with interview scores (Powell et al., 2018). It could be the case that reduced use of honest self-promotion tactics could be one mechanism through which interview anxiety is associated with poorer interview performance.

An additional area for future research could be investigating honesty-humility and extraversion and their relations with specific facets of interview anxiety, and also with different types of IM. For example, it appears that extraversion is negatively related to all five facets of interview anxiety (*r* ranging from − 0.23 to − 0.39). In contrast, honesty-humility has a very small correlation with behavioral anxiety (*r* = − 0.05) and a stronger relation with performance anxiety (*r* = − 0.18). Honesty-humility also has different relations with the types of IM (e.g., *r* = − 0.26 with image protection and *r* = − 0.16 with extensive image creation and deceptive ingratiation). It could be the case, for example, that people low in honesty-humility experience more performance anxiety, and subsequently engage in more image protection. We had no specific hypotheses about these relationships at the facet level, but future research could provide a deeper understanding of these relations by investigating facets of both interview anxiety and deceptive IM. This fine-grained approach to looking at the antecedents of honest and deceptive IM would certainly help better understand the role of individual differences in the use of such tactics. This research would contribute to refining the theoretical underpinnings of IM in interviews, and more broadly in personnel selection as a whole.

Consistent with this idea, there may be merit to exploring other potential ways that personality, anxiety, and IM relate to one another. For example, it could be that in addition to certain individuals being more likely to experience interview anxiety, those with certain traits may be more or less likely to react by faking when they are experiencing anxiety. Perhaps, anxiety may generally lead to more faking, but may be particularly likely to do so for people who may feel more capable of faking. This implies an interaction between traits and anxiety in predicting deceptive IM, in addition to a main effects model. Although not hypothesized, in the present study, we tested whether extraversion and honesty-humility interacted with overall interview anxiety to predict overall deceptive IM. Although we did not find these interactions to be significant, it may be that future studies utilizing more faceted a priori predictions, or a broader array of personality traits, could explore this possibility.^4^

### Implications

This research adds to our understanding of motivations underlying deceptive IM. Deceptive IM is positively correlated with the experience of anxiety during the interview, and it may be the case that both of these variables are related to concerns over self-presentation during the interview. Indeed, the interview contains all the elements that make social situations threatening, including being the focus of others’ attention and being judged by others. Interviews are different in this regard from other types of selection tools, and thus, social anxiety as an underlying cause for IM may be unique to interviews. If interview anxiety is an antecedent to deceptive IM, then a practical implication would be that interventions that reduce interview anxiety could also decrease applicants’ use of deceptive IM. In turn, this would presumably allow organizations to get a more accurate assessment of their candidates. It would be interesting, in future field research, to see if putting job candidates at ease prior to an interview would indeed reduce both their interview anxiety and their motivation to engage in deceptive IM in the interview. Currently, there is little research investigating factors that might put interviewees at ease; existing work (e.g., McCarthy & Cheng, 2014) has focused mainly on techniques that interviewees themselves can engage in. Tross and Maurer (2008) found that a coaching intervention that increased interviewee knowledge about the specific type of interview and what is expected to be successful in answering these types of questions was successful in improving interview performance. Although a full coaching intervention is not likely to be feasible for organizations, perhaps increasing the background material provided to candidates at the start of the interview (e.g., the types of questions they are going to be asked) may reduce their feelings of uncertainty and resulting anxiety about the interview. Such field research would contribute to our understanding of the causal relationship between interview anxiety and deceptive IM and would allow organizations to refine their personnel selection approaches.

## Electronic Supplementary Material

ESM 1 (DOCX 13 kb)

## Footnotes

1 Note that despite similar names, the social dimension of interview anxiety as defined by McCarthy and Goffin (2004) is narrower in scope than the broad category of social anxieties discussed by Schlenker and Leary (1982).

2 In addition to deceptive IM, we also measure honest IM tactics. However, we do not make specific hypotheses about how honest IM might relate to interview anxiety.

3 The data from the 2017 sample have been published, answering different research questions (see Amaral, Powell, & Ho, 2019). Additional information is provided in the data transparency appendix in the supplementary materials.

4 We should note, however, that while the interaction term between IM was highly non-significant (*p* = .91), the interaction between honesty-humility and interview anxiety was marginally significant (*p* = .09), and so there may be more merit to exploring that relationship further. That being said, anxiety was still significantly related to deceptive IM at ± 1 SD levels of honesty-humility, indicating that although the relationship between anxiety and deceptive IM may vary based on one’s honesty-humility (if we take the marginally significant finding as meaningful), the relationship is still significant at high and low levels of honesty-humility.
