# Supplementary material for: Shake and Fake: the Role of Interview Anxiety in Deceptive Impression Management
Source: J Bus Psychol. 2020 Aug 7;36(5):829–40. doi: 10.1007/s10869-020-09708-1 (PMC8550053; doi:10.1007/s10869-020-09708-1)
Supplement: Supplementary file 1 — (DOCX 13 kb) [file 10869_2020_9708_MOESM1_ESM.docx]

**Appendix – Data Transparency**

| Variables | **Current paper** (includes 123 participants from 2017 and 79 participants from 2018) | **Published Paper #1 (Amaral et al., 2019)**  (includes the 123 participants from 2017) |
| --- | --- | --- |
|  |  |  |
| Slight Image Creation | Yes | Yes |
| Extensive Image Creation | Yes | Yes |
| Deceptive Ingratiation | Yes | Yes |
| Image Protection | Yes | Yes |
| Honest Self Promotion | Yes | Yes |
| Honest Ingratiation | Yes | Yes |
| Honest Defensive | Yes | No |
| Interview Anxiety – Communication | Yes | No |
| Interview Anxiety – Appearance | Yes | No |
| Interview Anxiety – Social | Yes | No |
| Interview Anxiety – Performance | Yes | No |
| Interview Anxiety - Behavioral | Yes | No |
| Honesty-Humility | Yes | No |
| Extraversion | Yes | No |
